# Indoor air pollution from biomass fuel smoke is a major health concern in the developing world

**DOI:** 10.1016/j.trstmh.2008.05.028

**Published:** 2008-09

**Authors:** Duncan G. Fullerton, Nigel Bruce, Stephen B. Gordon

**Affiliations:** a Liverpool School of Tropical Medicine, Pembroke Place, Liverpool L3 5QA, UK; b Malawi-Liverpool-Wellcome Trust, Clinical Research Programme, PO Box 30096, Chichiri, Blantyre 3, Malawi; c Division of Public Health, University of Liverpool Whelan Building, Quadrangle, Liverpool L69 3GB, UK

**Keywords:** Fuels, Biomass fuel, Toxicity, Respiratory tract infection, Chronic obstructive pulmonary disease, HIV

## Abstract

One-third of the world's population burn organic material such as wood, dung or charcoal (biomass fuel) for cooking, heating and lighting. This form of energy usage is associated with high levels of indoor air pollution and an increase in the incidence of respiratory infections, including pneumonia, tuberculosis and chronic obstructive pulmonary disease, low birthweight, cataracts, cardiovascular events and all-cause mortality both in adults and children. The mechanisms behind these associations are not fully understood. This review summarises the available information on biomass fuel use and health, highlighting the current gaps in knowledge.

## 1 Introduction

**Figure 1:**
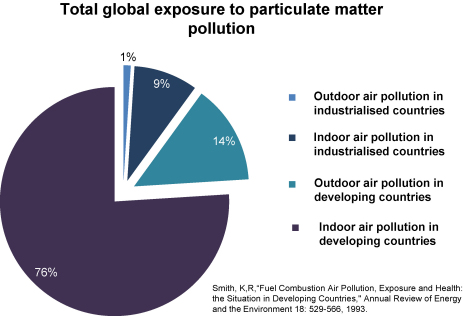
Pie chart showing total global exposure to particulate matter air pollution. Prepared using data from Smith (1993) by kind permission.

Air pollution is a significant cause of morbidity and mortality. The greatest health impacts from air pollution worldwide occur among the poorest and most vulnerable populations. The amount of exposure in terms of the number of people, exposure intensity and time spent exposed is far greater in the developing world (Smith, 1993); approximately 76% of all global particulate matter air pollution occurs indoors in the developing world (Figure 1).

When attention is focused on the problem of indoor air pollution resulting from the use of ‘biomass fuels’ (BMF), an enormous health burden is uncovered. There is now evidence linking an increased risk of respiratory tract infections, exacerbations of inflammatory lung conditions, cardiac events, stroke, eye disease, tuberculosis (TB), cancer and hospital admissions with air pollution levels (Atkinson et al., 1999, Hong et al., 2002, Laden et al., 2006, Lin et al., 2007, Pokhrel et al., 2005; Pope III et al., 2002; Saha et al., 2005, Smith et al., 2004). Data relating to the effects of burning BMF on health are of relevance to any physician practising in the developing world, however they are conspicuously underrepresented in the literature (Jaakkola and Jaakkola, 2006).

## 2 What is biomass fuel?

BMF refers to burned plant or animal material; wood, charcoal, dung and crop residues account for more than one-half of domestic energy in most developing countries and for as much as 95% in lower income countries (Smith et al., 2004). Around 2.4 billion people rely on BMF as their main source of domestic energy for cooking, heating and lighting (Reddy et al., 1996, Smith et al., 2004) and a further 0.6 billion people use coal. The adverse health effects of indoor air pollution are often exacerbated by lack of ventilation in homes using BMF and by the poor design of stoves that do not have flues or hoods to take smoke out of the living area. The combustion efficiency of BMF is also very low, thus it yields relatively high levels of products of incomplete combustion, which are more damaging to health.

The polluting effect, efficiency and cost of domestic fuel use are often construed as an ‘energy ladder’ (WHO, 2006a). Dried animal dung, scavenged twigs and grass, which are cheap, inefficient and pollute the most, are at the bottom of the ladder. Crop residues, wood and charcoal are a higher level BMF, whilst kerosene, coal and bottled or piped gas are the most efficient (non-BMF) combustible energy sources. Electricity is at the top of the energy ladder. The correlation of socioeconomic factors with the main fuel used is relatively close, however most households use several fuels in different settings. Four factors that appear to be most relevant in a household's choice of fuel type are: (a) cost of fuel, stove type and accessibility to fuels; (b) technical characteristics of stoves and cooking practices; (c) cultural preferences; and lastly, if at all, (d) the potential health impacts (Masera et al., 2000).

## 3 Toxic products in biomass smoke

Inefficient burning of BMF on an open fire or traditional stove generates large amounts of particulate matter as well as carbon monoxide, hydrocarbons, oxygenated organics, free radicals and chlorinated organics (Naeher et al., 2007). The particulate matter component of this smoke is classified according to its size, with inhalable material <10 μm in aerodynamic diameter referred to as PM_10_. The 24-h mean particulate matter levels set in the WHO guidelines for air quality are 50 μg/m^3^ for PM_10_ and 25 μg/m^3^ for PM_2.5_ (WHO, 2006b), but in many parts of the developing world the peak indoor concentration of PM_10_ often exceeds 2000 μg/m^3^ (Ezzati and Kammen, 2001, Regalado et al., 2006). Critically, there are age, gender and socioeconomic differences in levels of exposure and the consequent health effects (Bruce et al., 2000). Exposure to BMF has been estimated to have caused 0.5% of all deaths and 0.4% of all disability-adjusted life-years in South Africa in 2000 (Norman et al., 2007).

This review will examine the health effects on children and adults separately as well as distinguishing between respiratory and non-respiratory illness.

## 4 Respiratory illness in children

Young children living in households exposed to solid fuel (BMF) have a two to three times greater risk of developing acute lower respiratory tract infection (ALRI) compared with those living in households using cleaner fuels or suffering less exposure to smoke (Smith et al., 2000). In children under 5 years, the mortality attributable to ALRIs is estimated to be over 2 million deaths per year (Black et al., 2003, Murray et al., 2001, Rudan et al., 2004). The first report of indoor cooking smoke associated with childhood pneumonia and bronchiolitis was in Nigeria (Sofoluwe, 1968), however not until the 1980s was this followed by reports from other areas (Collings et al., 1990, Mtango et al., 1992, O’Dempsey et al., 1996, Pandey et al., 1985, Shah et al., 1994). One relatively small cohort study in rural Kenya found that the amount of pollution a child is exposed to directly correlates with the risk of developing pneumonia (Ezzati and Kammen, 2001).

Outdoor air pollution has chronic adverse effects on lung development in US children from the age of 10–18 years, which leads to clinically significant deficits in attained forced expiratory volume in 1 s (FEV_1_) as children reach adulthood (Gauderman et al., 2004). Carbon particles, similar to those found in ambient air and a biomarker of exposure to air pollution, are present in the airway macrophages of exposed children, and an increased level of carbon in their macrophages correlates with decreased lung function (Kulkarni et al., 2006). Data from Ecuador corroborate the deterioration in lung function expected when children are exposed to high levels of indoor air pollution from BMF (Rinne et al., 2006), and data from Guatemala have suggested that symptoms of wheeze are more frequent amongst households that use an open fire compared with a stove with a chimney (Schei et al., 2004). A review of data from China has shown reductions in children's FEV_1_, forced vital capacity and peak flow associated with domestic coal use (Zhang and Smith, 2007). However, as yet there are no longitudinal data on BMF exposure and children's lung function.

## 5 Non-respiratory illness in children

### 5.1 Low birthweight

**Figure 2:**
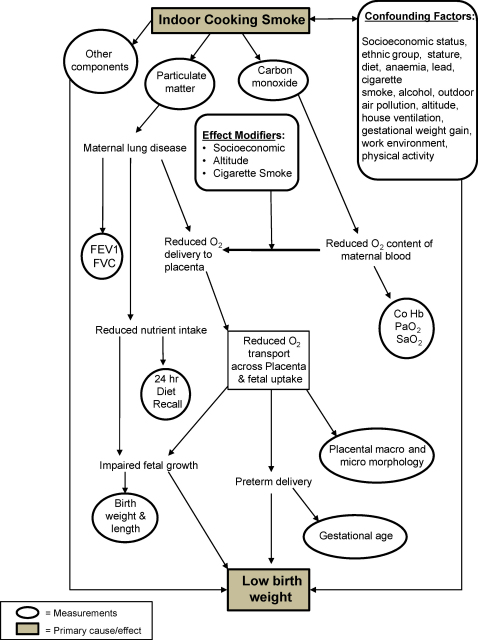
Pathways relating smoke exposure and childhood health. FEV1: forced expiratory volume in 1 s; FVC: forced vital capacity. Reproduced with kind permission of J.D. Haas: ‘Potential mechanisms for the effect of indoor cooking smoke on fetal growth’. Invited paper presented at WHO Workshop on ‘The Impact of Indoor Cooking Smoke on Health’, Geneva, Switzerland, 26–29 February 1992.

Evidence exists that implicates exposure to BMF smoke in adverse effects on different birth outcomes (Sram et al., 2005). There is a published association of low birthweight, intrauterine growth retardation and perinatal mortality with air pollution (Dejmek et al., 1999, Mavalankar et al., 1991, Wang et al., 1997). A study from Guatemala identified an association between birthweight and type of fuel used. The use of an open fire produced average levels of PM_10_ of 1000 μg/m^3^. The babies of mothers using open wood fires were on average 63 g lighter compared with babies born to mothers using cleaner fuels (Boy et al., 2002). A similar (slightly larger) effect has also been reported in Zimbabwe (Mishra et al., 2004). The model in Figure 2 attempts to explain how BMF may fit into a multifactorial explanation of low birthweight.

### 5.2 Nutritional deficiency

A recent report has suggested that exposure to BMF smoke in young children contributes to chronic nutritional deficiencies including anaemia and stunted growth (Mishra and Retherford, 2007). This was a study of nearly 30 000 children taking into account potential confounders such as exposure to tobacco smoke, recent episodes of illness, maternal education and nutrition, and other factors. However, the possibility of residual confounding still exists: the mix of fuels at different locations; biomass-exposed children are more likely to live in a rural area and to be in households of a lower standard of living and to live in lower-quality housing; and mothers are less likely to have received iron supplementation during pregnancy. Although Mishra and Retherford (2007) use multiple logistic regression models in an attempt to control for these factors, the extent to which confounding can be controlled depends on the accurate measurement of the full set of confounding factors and their inclusion in the specified model. The reliance on national survey data inherently limits confounder assessment. For example, there were no data on intensity of hookworm or other helminth infections, which are important causes of anaemia and malnutrition in this and other regions. These findings demonstrate a further set of outcomes from BMF exposure that require more investigation.

## 6 Respiratory illness in adults

Women bear the brunt of the disease burden associated with BMF, primarily because it is women living in rural areas who are exposed to high levels of BMF smoke. In Nepal, the average PM_10_ level in kitchens using BMF was three times higher than in those using cleaner fuels such as kerosene, liquefied petroleum gas (LPG) and biogas, and 94% of the respondents were disadvantaged women (Shrestha and Shrestha, 2005).

### 6.1 Interstitial lung disease

BMF smoke is associated with an interstitial lung disease referred to as ‘hut lung’ (Gold et al., 2000, Grobbelaar and Bateman, 1991), a form of pneumoconiosis in rural women from developing countries, originally described as ‘Transkei silicosis’ because it was thought to be due to silica particles. However, it is the contribution of BMF smoke in the pathogenesis of chronic obstructive pulmonary disease (COPD) that causes a greater global burden of disease.

### 6.2 Chronic obstructive lung disease

BMF smoke is responsible for COPD in non-smoking women living in rural areas (Ezzati, 2005, Orozco-Levi et al., 2006, Smith et al., 2004)*.* In women from rural Turkey it is estimated that the fraction of COPD attributed to exposure to biomass smoke, after adjusting for possible confounding factors, is 23.1% (Ekici et al., 2005). Cigarette smoking rates remain relatively low in developing countries compared with Europe and the USA (Muula, 2007, Rudatsikira et al., 2007). However, in Mexico, women exposed to domestic BMF smoke develop COPD with clinical characteristics, quality of life and increased mortality similar in degree to that of tobacco smokers (Ramirez-Venegas et al., 2006, Regalado et al., 2006).

### 6.3 Tuberculosis

Evidence is emerging that the incidence of TB is increased amongst BMF-exposed women. Studies from Mexico and India have implied a causal role of current BMF smoke exposure and the development of TB (Mishra et al., 1999, Perez-Padilla et al., 2001). Although these finding have not been seen in all studies, overall the evidence supports the hypothesis that exposure to respirable pollutants from combustion of BMF increases the risk of TB infection and disease (Lin et al., 2007). It is known that BMF smoke impairs alveolar macrophage function (Aam and Fonnum, 2007, Arredouani et al., 2006, Zhou and Kobzik, 2007). Alveolar macrophages are not only the target of *Mycobacterium tuberculosis* infection but also contribute an important early defence mechanism against bacteria. Therefore, it seems intuitive that BMF smoke also leads to an increased incidence of TB. However, more epidemiological and laboratory data are needed to support this hypothesis.

### 6.4 Lung cancer

Data from China imply that domestic coal smoke is a significant risk factor for the development of lung cancer (Du et al., 1996, Zhao et al., 2006). In studies from India and Mexico, data for non-smoking women exposed to BMF smoke for a number of years suggest that long-term exposure to BMF smoke from cooking may contribute to the development of adenocarcinoma of the lung (Behera and Balamugesh, 2005, Hernandez-Garduno et al., 2004). The International Agency for Research on Cancer (IARC) recently termed biomass smoke a ‘probable carcinogen’ (Group 2a) and coal (used as domestic fuel) was termed carcinogenic to humans (Group 1) (Straif et al., 2006).

## 7 Non-respiratory illness in adults

### 7.1 Cardiovascular disease

Particulate air pollution is statistically and mechanistically linked to increased cardiovascular disease (Brook et al., 2004). Long-term prospective cohort studies show an association between levels of air pollution consisting of fine particulate matter (PM_2.5_) and an elevated risk of death from all causes and from cardiovascular disease (Dockery et al., 1993; Pope III et al., 1995). More recent data have shown that non-fatal ischaemic events are also associated with an increase in fine particulate concentrations in the community (Miller et al., 2007). There is a paucity of data on the association between cardiovascular disease and BMF, but it is known that particulate air pollution leads to rapid and significant increases in fibrinogen, plasma viscosity, platelet activation and release of endothelins, a family of potent vasoconstrictor molecules (Brook et al., 2004). Recently, biomass smoke in Guatemalan women has been shown to increase diastolic blood pressure (McCracken et al., 2007). Therefore, it is highly likely that BMF represents a considerable risk to cardiovascular health. If the risks from outdoor air pollution are translated to BMF use, the number of premature deaths globally will be large—approaching 4% of the total global burden of disease (Smith, 2002).

### 7.2 Cataracts

The prevalence of cataracts is high in developing countries (Lewallen and Courtright, 2002). Epidemiological studies from Nepal and India have associated indoor cooking using BMF with cataracts or blindness (Pokhrel et al., 2005, Saha et al., 2005). Smoke induces oxidative stress and depletes plasma ascorbate, carotenoids and glutathione, which provide antioxidant protection against cataract formation. In a large, 89 000-household, Indian national survey, an adjusted odds ratio of 1.3 for blindness in women was found in homes using BMF, even after correction for a wide range of potentially confounding socioeconomic factors.

## 8 Could biomass fuel use exacerbate the health effects of HIV infection?

Globally, HIV now affects 30 million adults (Steinbrook, 2004). In parts of the world where HIV infection is most common, BMF is the main energy source. In Malawi, for example, the incidence of HIV in pregnant women is 33%, and 70% of hospital admissions (Lewis et al., 2003, Taha et al., 1998) and >80% of households use BMF. However, the influence of BMF smoke on HIV-infected individuals has not been clarified.

The most important effect of HIV infection in Africa is to cause increased bacterial infections, pneumonia and TB. HIV infection is associated with mild airway obstruction and loss of gas transfer, with severe impairment occurring in the presence of *Pneumocystis jiroveci* infection (Mitchell and Clarke, 1995). HIV is also associated with the accelerated development of COPD (Diaz et al., 2003) and it is likely, although not proven, that HIV infection is a significant contributor to airway disease in much of the adult population of Africa. Since both BMF use and HIV are associated with an increase in the incidence of pneumonia (Gordon et al., 2002, Smith et al., 2000) and as particulate matter exposure and HIV result in increased pulmonary inflammation (Ghio et al., 2000, Rowland-Jones, 2003, van Eeden et al., 2001), it is possible that by causing pulmonary inflammation the two major risk factors for pneumonia in African adults (HIV and BMF smoke) may actually demonstrate previously unrecognised synergy.

## 9 Intervention strategies and areas for future study

Environmentalist concerns about deforestation have driven the development of many new types of cooking stove that either have increased efficiency or use less polluting fuels (http://www.esmap.org/). The lower smoke output observed with these stoves has allowed health professionals to use them in trials as health interventions. However, systematic evaluations have shown that there are practical barriers to stove adoption (Barnes et al., 1994). The technical complexities of stove design, lack of maintenance and users’ behaviour, which modify ideal combustion, have also led to highly variable stove performance in everyday use compared with laboratory testing.

To be effective, interventions must take into account specific local conditions such as variations in the natural environment and climate, the purposes of energy use (e.g. cooking vs. heating), local infrastructure, user behaviours and sociocultural circumstances. For instance, changes in housing and having a separate kitchen or additional windows can reduce exposure, although reductions are likely to be smaller for those who cook and remain close to their fires. Moreover, burning fuel more cleanly by pre-processing it may be appropriate depending on geographical location, e.g. using charcoal in parts of sub-Saharan Africa or biogas in parts of Asia.

**Figure 3:**
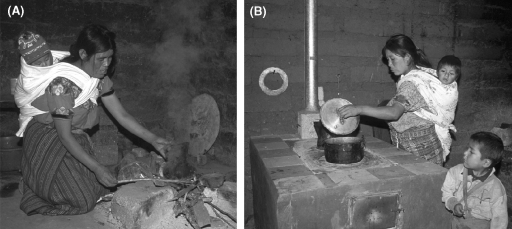
(A) Traditional open fire and (B) plancha stove. Images by Nigel Bruce.

The first randomised controlled trial performed on the health effects of solid fuel use in Guatemala, using the ‘plancha’ chimney stove (Figure 3A and B), was recently reported (Diaz et al., 2007). This challenging fieldwork revealed that exposure to smoke, measured using exhaled carbon monoxide as a surrogate marker, was reduced with the plancha, as were symptoms of sore eyes and back pain. However, as yet there are no published spirometric, birthweight, ALRI rate or other health data.

Current understanding suggests that lower emissions will be more effectively achieved in the poorest communities by modifying specific aspects of current fuel stove and energy use behaviours rather than by attempting to replace the solid-fuel stoves with stoves that use liquid fuel, gas or electricity. For communities that already purchase some or all of their BMF and where supply of clean fuels is (or could become) cheaper and more reliable, then development initiatives to support a switch to LPG or other liquid or gaseous fuels has a higher chance of success.

Table 1 shows the nature of possible solutions to reducing BMF smoke (Bruce, 2005). Given the number of potential strategies and the wide range of agencies responsible for these interventions, it may be thought that health services have little or no role in addressing this problem. Health services might be seen as being at the receiving end of the consequence of biomass smoke but not in a good position to do anything effective about it.

**Table 1:** Potential interventions to reduce exposure to indoor air pollution

Source of pollution	Living environment	User behaviours
Improved cooking devices:	Improved ventilation:	Reduced exposure through operation of source:
• Improved biomass stoves	• Hoods, fireplaces, chimneys, built into the structure of the house	• Fuel drying
• Improved stoves with flues attached	• Windows, ventilation holes, e.g. in roof, which may have cowls to assist extraction	• Use of pot lids to conserve heat
		• Good maintenance of stoves, chimneys and other appliances

Alternative fuel cooker combinations:	Kitchen design and placement of the stove:	Reductions by avoiding smoke:
• Briquettes and pellets	• Kitchen separate from house reduces exposure of family (less so for cook)	• Keeping children away from smoke, e.g. in another room (if available and safe to do so)
• Charcoal	• Stove at waist height to reduce direct exposure of cook leaning over fire	
• Kerosene		
• Liquid petroleum gas		
• Biogas, producer gas		
• Solar cookers (thermal)		
• Other low-smoke fuels		
• Electricity		

Reduced need for the fire:		
• Insulated fireless cooker (haybox)		
• Efficient housing design and construction		
• Solar water heating		

*Source*: Bruce (2005). © by FSG Communications Ltd. Reproduced with kind permission.

However, this would miss the important input that health professionals can have. In their contact with patients with pneumonia, COPD and other health issues, health professionals can assess the risks, raise awareness and provide guidance on reducing exposure. Public health education and ‘brief interventions’ by clinicians have been shown to have a significant impact on disease burden (Rigotti et al., 2007). This topic also provides important opportunities for clinical and epidemiological research, the findings of which can be very influential within a country. Those within the health system responsible for planning and management can make good use of information from healthcare and local research, and contribute to awareness-raising through the media and educational activities, as well as lend their voices to calls for action at local, national and international forums.

## 10 Conclusions

Indoor air pollution from BMF disproportionately affects women and children and is the cause of significant global mortality and morbidity. This is a neglected area of global disease that affects a large proportion of the world's population. The most pressing areas of research are:

• toxicological studies to help plan appropriate intervention studies;
• exposure assessment tools and biomarkers to aid epidemiological study;
• epidemiological studies to examine the effect on birth outcomes, TB, cancer and cardiovascular disease and the interaction of BMF with HIV; and
• randomised trials to test the effect of technological and behavioural interventions.

## Funding

This work is funded by the Wellcome Trust (Ref. 080065).

## Conflicts of interest

None declared.

## Ethical approval

Not required.
